# Identification of TgENT1 as the TgUUT1 Uracil/Uridine Transporter of *Toxoplasma gondii*

**DOI:** 10.3390/pathogens15030266

**Published:** 2026-03-02

**Authors:** Hamza A. A. Elati, Mariana Ferreira Silva, Lilach Sheiner, Harry P. de Koning

**Affiliations:** 1 School of Infection and Immunity, College of Medical, Veterinary and Life Sciences, University of Glasgow, Glasgow G12 8TA, UK; hamza.elati@glasgow.ac.uk (H.A.A.E.);; 2 Centre for Parasitology, School of Infection & Immunity, Sir Graeme Davies Building, University of Glasgow, Glasgow G12 8TA, UK; 3 Department of Pharmacology and Toxicology, Pharmacy College, University of Elmergib, Al Khums 40414, Libya

**Keywords:** *Toxoplasma gondii*, nucleoside transporter, uridine transport, 5-fluorouracil, pyrimidine antimetabolite

## Abstract

The protozoan pathogen *Toxoplasma gondii* is responsible for toxoplasmosis, a disease that can be deadly in immunocompromised patients and the developing fetus during pregnancy. Current treatments are widely considered to be suboptimal. We have recently reported that 5-fluoropyrimidines have highly promising anti-toxoplasmosis effects and are internalized by the parasite by a high-affinity uracil/uridine transporter, TgUUT1. Here, we attempt to identify the gene encoding this transport protein. The only nucleoside or nucleobase family identified in the *T. gondii* genome was the Equilibrative Nucleoside Transporter (ENT) family, with four members. Of these, TgAT1 is known to be purine-specific, and deletion of the *TgENT2* and *TgENT3* genes, either separately or jointly, did not affect uridine transport or sensitivity to 5-fluoropyrimidines. In contrast, depletion of *TgENT1*, an essential gene, resulted in a significant reduction in the uptake of both uracil and uridine but not of the amino acid tryptophan. Moreover, expression of *TgENT1* in a *Leishmania mexicana* cell line with low endogenous uracil uptake rates significantly increased uracil uptake for these cells. We conclude that it is highly probable that *TgENT1* encodes the *T. gondii* uracil/uridine transporter. On the basis of our previous results, we infer that TgENT1 likely also mediates the uptake of 5-fluoropyrimidines.

## 1. Introduction

Toxoplasmosis is a common infection of humans and mammals that can be dangerous in its acute form, especially if contracted during pregnancy, as the pathology to the unborn baby can be severe and even fatal [1]. Moreover, chronic infection is thought to persist for life and poses a severe risk to immunocompromised patients [2]. Despite the clinical importance of toxoplasmosis, there are still numerous gaps in our knowledge of the biology of its etiological agent, *Toxoplasma gondii*.

*T. gondii* is an apicomplexan parasite and, like other Apicomplexa, including the *Plasmodium* parasites that cause malaria, it is an obligate intracellular parasite [3]. In its tachyzoite form, *T. gondii* is able to invade a broad range of nucleated cell types of almost any warm-blooded animal [4]. Despite its critical dependency on the host for survival, our understanding of how the parasite obtains essential nutrients such as nucleotides remains poor.

Like all other parasites, *T. gondii* express highly efficient salvage systems to obtain nutrients from the host environment. This involves the manipulation of nutrient uptake by the host cell [5,6]. Pores in the parasitophorous vacuole membranes allow the diffusion of these nutrients directly to the parasite [7,8], where they are internalized by an array of highly effective nutrient transporters that typically outcompete host transporters in substrate affinity and translocation efficiency.

*Toxoplasma* tachyzoites, like many other parasites, also have high replication rates, requiring large amounts of nucleotides for DNA and RNA synthesis, among other functions. Like all other protozoan parasites where this has been investigated, *Toxoplasma* lack the biosynthesis pathway for purines [9] and thus rely fully on transporters and salvage enzymes for these critical biochemicals. They cannot take up nucleotides like ATP directly from the host cytosol [10] and thus need particularly high-affinity transporters to salvage free nucleosides and nucleobases, which are present in low concentrations in the host environment. High-affinity transporters for purine nucleosides and oxopurine nucleobases have been described [11].

In contrast, *T. gondii* express the entire pathway for de novo pyrimidine synthesis, and although this is important for virulence, it is not essential for cellular invasion in vitro if the medium is supplemented with >20 µM uracil [12]. This constitutes functional evidence that *T. gondii* expresses a transporter capable of uracil salvage, and we recently confirmed that uracil and also uridine are indeed readily taken up by *T. gondii* tachyzoites [13] but that its capacity is insufficient to sustain in vivo replication.

In recent years, numerous nucleoside and nucleobase transporters of protozoan parasites have been cloned and characterized [14,15,16,17], and all were found to be members of the Equilibrative Nucleoside Transporter (ENT) family. Whereas members of the Concentrative Nucleoside Transporter (CNT) family are commonly found in mammalian hosts and many other species [18], none have been found in protozoan genomes. Despite this progress in the characterization of nucleoside and nucleobase transporters, particularly from kinetoplastids and *Plasmodium* species, our understanding of purine and pyrimidine salvage in *Toxoplasma* remains very incomplete.

Apart from their importance in the physiology of protozoan pathogens, transporters have potential as drug targets [19,20]. For *Toxoplasma*, this potential is strongly supported by our recent report showing that the 5-fluoropyrimidines 5-fluorouracil (5-FU), 5-fluorouridine (5F-Urd) and 5-fluoro,2′-deoxyuridine (5F,2′dUrd) are efficiently taken up by *T. gondii* tachyzoites and display 10-fold higher activity against intracellular parasites than the antifolate sulfadiazine, the “current gold standard treatment” when combined with pyrimethamine [21]. In a first pilot experiment, 5-FU was at least as effective as sulfadiazine in a mouse model of acute toxoplasmosis [13]. Moreover, 5-fluoropyrimidines have long been used as anticancer chemotherapy [22] and, according to the one report of their use against acute toxoplasmosis in AIDS patients in combination with the second-line toxoplasmosis drug clindamycin [23], would be safe to use as they are effective at one-tenth the dose used against cancer.

Although our previous report identified a new uracil/uridine transporter (TgUUT1) as responsible for the uptake of the 5-fluoropyrimidines, the gene encoding this carrier has not yet been identified. Indeed, although the *T. gondii* genome project has long since identified four likely nucleoside or nucleobase transporters of the ENT family (Table 1), only one of the four genes and its product has been characterized, Tg_244440, which was first identified from a strain made resistant to the adenosine analogue adenine arabinoside (Ara-A), as a low-affinity adenosine transporter, and the corresponding protein was therefore named TgAT1 [24]. Recently, we confirmed, by heterologous expression in *Trypanosoma brucei*, that TgAT1 is indeed a low-affinity transporter for adenosine but, more surprisingly, it also displayed much higher affinity for the oxopurine nucleobases hypoxanthine and guanine and their nucleosides inosine and guanosine [25]. Of the three remaining genes encoding putative ENT family members (here collectively called *TgENT*s), Tg_288540 is reportedly an essential gene for *T. gondii* in culture [26], and Tg_233130 is upregulated in bradyzoites [27].

Here, we start to address the issues raised above, including the role of the TgENTs in pyrimidine uptake, to try to identify the gene encoding TgUUT1, being the carrier for the 5-fluoropyrimidine antimetabolites.

## 2. Materials and Methods

### 2.1. In Vitro Culture of Host Cells and Parasites

#### 2.1.1. *T. gondii* in Human Fibroblasts

The F3 tomato strain (RH Δku80 TATi) [13,28], herein referred to as RH, was used as the primary cell line for generation of all the mutant cell lines and was also used as the control for all the biochemical experiments such as transport assays and drug screening assays, qRT-PCR and plaque assays.

*T. gondii* tachyzoites of the RH and mutant lines were cultured in human foreskin fibroblasts (HFF), sourced from ATCC (SCRC-1041). Parasites were passaged routinely as a confluent monolayer of HFF in Dulbecco’s Modified Eagle’s Medium (DMEM) containing 4.5 gL^−1^ glucose (Sigma, St Louis, MO, USA), supplemented with 10% (*v*/*v*) fetal bovine serum (Gibco, Life Technologies, Paisley, UK), 4 mM L-glutamine and penicillin/streptomycin (Life Technologies) antibiotics and grown at 37 °C with 5% CO_2_. Where mentioned, anhydrotetracycline (ATc; Sigma) was added to the medium at a final concertation of 0.5 µM.

#### 2.1.2. *Leishmania* Promastigotes

Three strains of *L. mexicana* promastigote forms were mainly used in this part of the project: (1) *L. mexicana-Cas9* T7 strain (derived from *L. mexicana* WT promastigotes by expression of the *Streptococcus pyogenes* Cas9 nuclease gene “Cas9” and maintained on 32 µg/mL hygromycin [29], generously donated by Prof. Eva Gluenz (University of Bern, Switzerland). (2) The *Lmex-NT3-KO* strain (∆NT3) was generated from the *L. mexicana Cas9* strain by CRISPR-mediated deletion of the NT3 transporter gene [30,31]. (3) The *Lmex-NT3-KO* strain was used as an expression system to study TgENT1. All *Leishmania* strains were grown as promastigotes, in standard HOMEM (GIBCO, Life Technologies) supplemented with 10% heat-inactivated fetal bovine serum (FBS; PAA Laboratories, Linz, Austria) and 1% of a penicillin–streptomycin solution (Life Technologies) at 25 °C, as described by Al-Salabi et al. [32].

### 2.2. Chemicals and Radiochemicals

Uridine, uracil, thymidine, cytidine, adenosine, inosine, 5-floururacil (5-FU), 5-flourouridine (5-FUrd), sulfadiazine, resazurin sodium salt and phenylarsine oxide (PAO) were sourced from Sigma-Aldrich (Poole, UK). 5-Flouro 2′-deoxyuridine (5-F-2′dUrd) was from Avantor (VWR, Radnor, PA, USA). [2,8-^3^H]-Adenine (40.3 Ci/mmol) was obtained from PerkinElmer (Waltham, MA, USA). [5,6-^3^H]-Uracil (40 Ci/mmol), [5,6-^3^H]-uridine (60 Ci/mmol), [2,8-^3^H]-adenosine (40 Ci/mmol) and [^3^H]-tryptophan (25 Ci/mmol) were sourced from American Radiolabeled Chemicals Incorporated (St Louis, MO, USA).

### 2.3. Drug Sensitivity Assay for T. gondii Tachyzoites

Drug sensitivity assays for *Toxoplasma gondii* tachyzoites were performed as previously described [13]. Briefly, HFF cells were seeded in 96-well black plates and grown to confluence. Test compounds and sulfadiazine (positive control) were prepared in DMEM and serially diluted across the plate, leaving the last column as a drug-free control. Freshly egressed tachyzoites (~1000/well) were added to each well and incubated for 6 days at 37 °C with 5% CO_2_. Fluorescence was measured using a PHERAstar plate reader (excitation: 540 nm; emission: 590 nm). EC_50_ values were calculated using GraphPad Prism 10.0 using a 4-parameter sigmoid curve (variable slope). All assays were performed in triplicate and repeated independently 3–5 times.

### 2.4. Drug Cytotoxicity Assay for HFF Cells Using Alamar Blue Dye

Cytotoxicity was as previously described [13], using PAO as the positive control. Plates were incubated for 6 days at 37 °C with 5% CO_2_. On day 6, 10 µL of resazurin solution (12.5 mg/100 mL ddH_2_O) was added to each well, including media-only wells for background fluorescence. After 3–4 h of incubation, fluorescence was measured (excitation: 540 nm; emission: 590 nm) using a PHERAstar plate reader. Data were analyzed in GraphPad Prism 10.0 using a four-parameter sigmoid curve to calculate EC_50_ values. Experiments were performed in triplicate and repeated 3–5 times.

### 2.5. Transport Assays

Transport of radiolabeled uridine, uracil and tryptophan into extracellular *T. gondii* tachyzoites and *L. mexicana promastigotes* was assayed following previously published protocols [11,13,16,31,33]. Briefly, *L. mexicana* promastigotes were grown at 27 °C for 40–48 h to mid-log phase, while *T. gondii* tachyzoites were harvested from confluent HFF cultures maintained at 37 °C and 5% CO_2_. Parasites were washed twice in assay buffer (AB: 33 mM HEPES, 98 mM NaCl, 4.6 mM KCl, 0.5 mM CaCl_2_, 0.07 mM MgSO_4_, 5.8 mM NaH_2_PO_4_, 0.03 mM MgCl_2_, 23 mM NaHCO_3_, 14 mM D-glucose; pH 7.3), counted and resuspended at 1 × 10^8^ cells/mL (*Leishmania*) or 2 × 10^8^ cells/mL (*T. gondii*). After 30 min of recovery at room temperature, 100 µL of cell suspension was layered over an oil mixture (1:7 mineral oil:di-*n*-butyl phthalate for *L. mexicana*, 1:5 for *T. gondii*) containing radiolabeled substrate. Incubations were performed for predetermined times and stopped by adding 750 µL of ice-cold stop solution (0.5–2 mM unlabeled substrate). Tubes were centrifuged at 14,800× *g* for 1 min, flash-frozen, and cell pellets were processed for scintillation counting after SDS lysis. All assays were done in triplicate and repeated in three independent experiments.

### 2.6. Plasmid Construction and Expression of TgENT1 in L. mexicana NT3-KO

The *TgENT1* gene was expressed in *L. mexicana* NT3-KO promastigotes [31] using the pNUS-HcN plasmid, which carries a neomycin resistance marker (G-418) [34]. The pNUS-HcN vector was digested with *NdeI* and *XhoI* according to the manufacturer’s protocol. *TgENT1* was amplified by PCR using Phusion High-Fidelity DNA Polymerase (Supplemental Tables S1 and S2). PCR products and digested plasmids were run on 1% agarose gels, visualized under UV light, and purified using the NucleoSpin PCR and Gel Extraction Kit (Macherey-Nagel; Thermo Fisher Scientific, Oxford, UK).

Ligation was performed using the NEBuilder HiFi DNA Assembly Kit (New England Biolabs, Ipswich, MA, USA). The reaction (10 µL total volume) contained 60 ng of digested pNUS-HcN vector and 180 ng of *TgENT1* insert (vector:insert ratio of 1:3), combined with 5 µL of NEBuilder HiFi Master Mix and nuclease-free water. The mixture was incubated at 50 °C for 3–4 h. The assembled plasmid was transformed into NEB 5-alpha competent *E. coli* by heat shock. Colonies were selected on LB agar containing 100 µg/mL of ampicillin, cultured in LB broth, and plasmid DNA was extracted using the NucleoSpin Plasmid Purification Kit.

Positive clones were screened by Phusion PCR using a *TgENT1*-specific forward primer (HDK-1739) and a pNUS-HcN reverse primer (HDK-340) (listed in Table S3). Confirmation was also achieved by restriction digestion with *NdeI* and *XhoI,* followed by Sanger sequencing (Source Bioscience, Livingston, UK) using M13F and gene-specific reverse primers (HDK-1740) (listed in Table S3). Sequence data were analyzed using CLC Genomics Workbench v7.0 (Qiagen, Hilden, Germany). Verified plasmids (pHDK295 “TgENT1”) were ethanol-precipitated, and 25 µg of DNA was resuspended in 15 µL of sterile water.

Transfection was performed as described [31]. Briefly, 5 × 10^7^ *L. mexicana* NT3-KO promastigotes were harvested, washed twice in PBS, and resuspended in 100 µL transfection buffer. Cells were mixed with 10 µg of circular pHDK295 plasmid DNA (episomal expression) or RNase-free water (negative control), transferred to a 0.2 cm cuvette, and electroporated using an Amaxa Nucleofector (Program U-033; Amaxa, London, UK). Cells were recovered overnight in HOMEM medium supplemented with 10% FBS at 25 °C, followed by selection with 50 µg/mL G-418. Cultures were plated by limiting dilution (1:10, 1:25, 1:100) in 96-well plates and incubated at 25 °C for 10–14 days to obtain individual clones. Positive clones were expanded in HOMEM medium containing G-418. Genomic DNA was extracted using the NucleoSpin Tissue Kit (Macherey-Nagel), and integration was confirmed by PCR using *TgENT1*-specific forward (HDK-1739) and pNUS-HcN reverse primers (HDK-340).

### 2.7. CRISPR-Mediated Gene Disruption in T. gondii Tachyzoites

#### 2.7.1. Direct Gene Knockout

Plasmids for gene knockout and knockdown were constructed using CRISPR-guided promoter replacement in *T. gondii* RH tomato ΔKu80 TATi cells (herein called RH cell line) as described previously [28,35]. Three plasmids were used for these strategies: (i) pDTS4-DHFR for non-essential gene replacement or promoter replacement for essential genes [28]; (ii) an mNeonGreen cassette plasmid for homologous recombination knockout [36]; and (iii) pg474 (Tub-Cas9-YFP-pU6-ccdB-tracrRNA) for delivery of sgRNA/Cas9 [35]. The pDTS4-DHFR plasmid was used to generate two single knockout strains of *TgENT2* and *TgENT3* and knockdown of *TgENT1*, while the mNeonGreen cassette was used to generate a double knockout (targeting *TgENT2* in a Δ*TgENT3* background) and Δ*TgAT1* in the RH strain.

Single-guide RNAs targeting the start codon region of TgAT1, TgENT1, TgENT2, and TgENT3 were designed using the ChopChop online website tool (https://chopchop.cbu.uib.no/ (accessed 15 May 2022) and are listed in (Supplemental Table S4). Each sgRNA was cloned into pg474 via *Bsa*I restriction sites. Plasmids were purified using Qiagen Miniprep and Midiprep kits according to the manufacturer’s instructions. The DHFR cassette allowed selection for pyrimethamine resistance [28], while mNeonGreen-positive parasites were selected by fluorescence-activated cell sorting (FACS). PCR primers for cassette amplification and diagnostic PCR are listed in Supplemental Table S5. Homology regions for DHFR cassettes were 50 bp at the 5′ and 3′ UTRs of the target gene, while mNeonGreen cassettes used 40 bp homology regions. PCR products were confirmed by agarose gel electrophoresis and purified using QIAquick PCR Purification Kit (Qiagen) prior to transfection.

#### 2.7.2. Gene Knockdown (Tetracycline-Inducible Transactivator System)

Gene knockdown constructs were generated using the same CRISPR/Cas9 workflow described above for direct gene knockout, with modifications to incorporate the ATc-repressible T7S4 promoter. Single-guide RNAs targeting the start codon region of *TgENT1* were designed using the ChopChop online website tool and are listed in Supplemental Table S1. Each sgRNA was cloned into pg474 via *Bsa*I restriction sites as described for knockout. The DHFR/T7S4 cassette was amplified from pDTS4-DHFR using primers bearing 50 bp homology to the *TgENT1* locus (Supplemental Table S5). PCR reactions were performed with Platinum SuperFi II DNA Polymerase (Thermo Fisher) (Supplemental Tables S6 and S7), and products were verified by agarose gel electrophoresis and purified using the QIAquick PCR Purification Kit (Qiagen). Plasmids were prepared using Qiagen miniprep/midiprep kits according to the manufacturer’s instructions.

#### 2.7.3. Transfection and Selection

Transfections were carried out using freshly egressed tachyzoites. Briefly, 1–5 × 10^6^ parasites were resuspended in Cytomix (120 mM KCl, 0.15 mM CaCl_2_, 25 mM HEPES, 5 mM MgCl_2_, 10 mM K_2_HPO_4_/KH_2_PO_4_, pH 7.6) supplemented immediately before use with 65 µL of 3 mM ATP and 65 µL of 3 mM glutathione. Approximately ~50 µL of purified PCR cassette and 50–70 µg of sgRNA/Cas9 plasmid were added (total electroporation volume ~0.8 mL in a 4 mm cuvette). Electroporation was performed using a square-wave protocol (1700 V, 0.2 ms pulse length, two pulses separated by 5 s). Parasites were transferred to confluent HFF monolayers and incubated at 37 °C with 5% CO_2_. Integration events using DHFR were selected with pyrimethamine (Sigma-Aldrich) (1 μM) within 24 h for 5–8 days. Once the drug-selected parasites grew out (5–7 days), a 96-well plate of HFF cells was used for cloning the pool by serial dilution. Correct integration and promoter replacement were verified by diagnostic PCR (Supplemental Tables S8 and S9) using the primers listed in Table S5; knockdown efficiency was assessed by qRT-PCR where indicated using the primers listed in Table S10.

#### 2.7.4. Fluorescence-Activated Cell Sorting

For constructs incorporating mNeonGreen (e.g., enrichment of double knockout pools and ΔTgAT1 pool), transfected parasites were allowed to recover for ~48 h and then released and passed through a 26 G needle and a 3 μm filter. The parasites were collected and centrifuged for 10 min at RT. The supernatant was discarded and resuspended in FACS buffer (1% fetal calf serum, 1 mM EDTA in PBS) at roughly 10^7^/mL and 1–2 mL transferred to FACS tubes. The transfected samples, including positive control (green) and negative control samples (red tomato), were sorted on a high-speed cell sorter (BD FACSAria IIu or BD FACSAria III high-speed cell sorter (BD Biosciences, Franklin Lakes, NJ, USA). Gating was established using non-transfected (RH) and mNeonGreen-positive controls. Single fluorescent parasites were sorted directly into 96-well plates pre-seeded with HFFs and cultured at 37 °C/5% CO_2_ for 5–7 days to obtain single clonal plaques. Single clones were confirmed by PCR and qRT-PCR using the primers listed in Tables S5 and S10.

### 2.8. Plaque Assay

Plaque assays were performed to assess parasite growth as described previously [37]. Confluent HFF monolayers in 6-well plates were infected with freshly egressed tachyzoites (100 parasites per well). Parasites were grown in the presence or absence of anhydrotetracycline (ATc, 0.5 µM) and incubated at 37 °C with 5% CO_2_ for 7 days without agitation. After incubation, wells were examined by light microscopy to confirm plaque formation. Cells were fixed by adding 300 μL of ice-cold 100% methanol per well and incubating for 20 min at room temperature. Fixed monolayers were washed three times with PBS and stained with 2 mL of 0.4% crystal violet solution for ~2 h at room temperature. Wells were washed three times with PBS, air-dried, and plaques were imaged.

### 2.9. Quantitative Real-Time PCR (qRT-PCR) in T. gondii Tachyzoites

qRT-PCR was carried out as previously described [38,39] to assess gene expression changes. Specifically, qRT-PCR was used to measure the expression level of mRNA for TgENT1 downregulation upon ATc treatment to confirm the deletion of *TgAT1, TgENT2, TgENT3,* and the double knockout (DK) lines and to evaluate potential upregulation or downregulation of TgENT genes in each knockout line. qRT-PCR primers were designed using the NCBI Primer-BLAST website Primer designing tool (Supplemental Table S10). For knockdown experiments, parasites were grown in the presence or absence of ATc for 24, 48, or 72 h; for knockout lines, parasites were grown without ATc. Parasites were collected, passed through a 3 µm polycarbonate filter, and pelleted by centrifugation at 1500× *g* for 10 min at RT. Total RNA was extracted using the RNeasy Mini Kit (Qiagen) with on-column DNase I treatment (Thermo Fisher) according to the manufacturer’s instructions. cDNA was synthesized using the High-Capacity RNA-to-cDNA Kit (Applied Biosystems). qRT-PCR reactions were set up with Power SYBR Green PCR Master Mix (Applied Biosystems) using 10 ng of cDNA as template and gene-specific primers (listed in Table S10). Reactions were run on a 7500 Real-Time PCR System (Applied Biosystems) under standard cycling conditions. Relative expression was calculated using the 2^−ΔΔCt^ method [40], using catalase mRNA as the internal control. Three independent biological replicates were performed for each condition, and data were analyzed and plotted using GraphPad Prism 10.0.

### 2.10. qRT-PCR for L. mexicana Promastigotes

qRT-PCR was performed as previously described [31,41] to determine the expression levels of TgENT1 in Lmex-NT3-KO compared to the control line and to select the optimal clone. RNA was extracted from *L. mexicana* promastigotes (2 × 10^6^ cells/mL) using the NucleoSpin RNA kit (Macherey-Nagel, Düren, Germany) according to the manufacturer’s instructions and quantified using a NanoDrop ND-1000 spectrophotometer. Samples were stored at −80 °C until further use. qRT-PCR primers were designed using NCBI Primer-BLAST website Primer designing tool (Supplemental Table S10). cDNA was synthesized from 2 µg of RNA using the Precision nanoScript II Reverse Transcription kit (PrimerDesign Ltd., Eastleigh, UK) following the manufacturer’s protocol and stored at −20 °C. Primer efficiency was determined using the Pfaffl method [42]. Amplification was performed using the PrecisionPLUS OneStep RT-qPCR Master Mix kit (PrimerDesign Ltd., Eastleigh, UK) with SYBR Green on a 7500 Real-Time PCR System (Applied Biosystems, Thermo Fisher Scientific). Cycling conditions were: 50 °C for 2 min, 95 °C for 10 min, followed by 45 cycles of 95 °C for 15 s and 60 °C for 1 min. A dissociation curve was included to confirm single-product amplification. Negative controls included samples without reverse transcriptase or cDNA. Gene expression was normalized to GPI8, a constitutively expressed housekeeping gene in *L. mexicana* [43]. Relative quantification was calculated using the 2^−ΔΔCt^ method, and data analysis was performed using Applied Biosystems 7500 Fast Real-Time PCR System Software. Each experiment was carried out in triplicate with three independent biological replicates, and data were analyzed and plotted using GraphPad Prism 10.0.

## 3. Results

### 3.1. Creation of TgENT Knockout (KO) and Knockdown (KD) Strains

#### 3.1.1. Identification of Toxoplasma ENTs

We performed protein–protein BLAST or position-specific iterated BLAST on ToxoDB (https://toxodb.org/toxo/app; accessed 15 March 2022) using human (ENT1-4, CNT1-3) or *Plasmodium falciparum* (ENT1-4) nucleoside transporter genes as query sequences to search for *Toxoplasma* homologs. Consistent with similar queries of other protozoan genomes [14,44], no homologs of human CNTs were identified in any of the genomes accessible through ToxoDB (https://toxodb.org/toxo/app; accessed 15 March 2022). However, the searches identified four genes in the *Toxoplasma* genome with ENT homology, which are numbered as follows in the ME49 genome: TGME49_233130, TGME49_244440, TGME49_288540, and TGME49_500147. Of these, gene TGME49_244440 was previously proposed to be an adenosine transporter (TgAT1) [24], and we propose to keep that name so as not to confuse the literature, although we have shown that this gene encodes a transporter with much higher affinity for oxopurines than for adenosine [25]. The other genes were given an NT1–NT3 numbering in the PhD thesis by Kshitiz Chaudhary of the research group of David Roos [45], and we propose, again, to keep the same numbering but name the genes *TgENT1–3* (Table 1), as the function of these genes has not been established yet. While they are likely members of the ENT gene family, nucleoside transporter activity was not demonstrated at this point. This naming is also consistent with a recent paper by Messina, Goerner, Bennett, Brennan, Carruthers and Martorelli Di Genova [26], and while a paper describing the cellular localization of some of the *T. gondii* nucleoside transporters [46] provided slightly different names, we propose to consolidate according to the current majority here (Table 1).

**Table 1:** ENT genes.

*T gondii* Gene ID		Name	bp (No Introns)	a.a.	TMD *	Qian et al. [46]
TGME49	TGGT1					
244440	244440	*TgAT1*	1389	462	10	*TgAT1*
288540	288540	*TgENT1*	2091	696	10	*TgNT1*
500147	359630	*TgENT2*	2007	668	10	*TgNT3*
233130	233130	*TgENT3*	1596	531	10	*TgNT2*

Data according to ToxoDB release 68, accessed 10 December 2025. *, https://services.healthtech.dtu.dk/services/TMHMM-2.0/ (accessed on 26 February 2026).

#### 3.1.2. Construction of *TgENT2* and *TgENT3* Knockouts in *T. gondii* RH Cell Line

In order to generate the tools to identify which one of the *TgENT* genes may encode the previously described uracil/uridine transporter TgUUT [13], we opted to use genetic deletion of *TgENT2* and *TgENT3* in the *T. gondii* RH background. Likewise, we targeted *TgENT1* where all attempts at creating a direct knockout were futile, as predicted in the CRISPR/Cas9 screen [47], indicating that *TgENT1* is essential for growth in culture, and a conditional knockdown was thus attempted instead; the other three *TgENT*s were found to be non-essential in the same screen and thus direct knockout (KO) was attempted. Finally, although we have previously characterized TgAT1 in detail and showed that it is not fully inhibited by even 1 mM uracil or uridine [25], the gene encoding this transporter was also targeted for deletion: (1) in order to confirm the previous observations regarding uracil and uridine, (2) the transporter displayed higher affinity for thymidine than for uracil, and 5F-uracil is really a thymine analogue, i.e., it is just possible that TgAT1 could take up 5-fluorinated uracil and uridine even though it does not recognize uracil and uridine itself, and (3) to examine the possibility that even if TgAT1 only displays low affinity and a slow translocation rate for 5-fluoropurimidines, this still could be sufficient for a parasiticidal effect if the parasite is exposed to a drug for a prolonged period [6].

**Figure 1:**
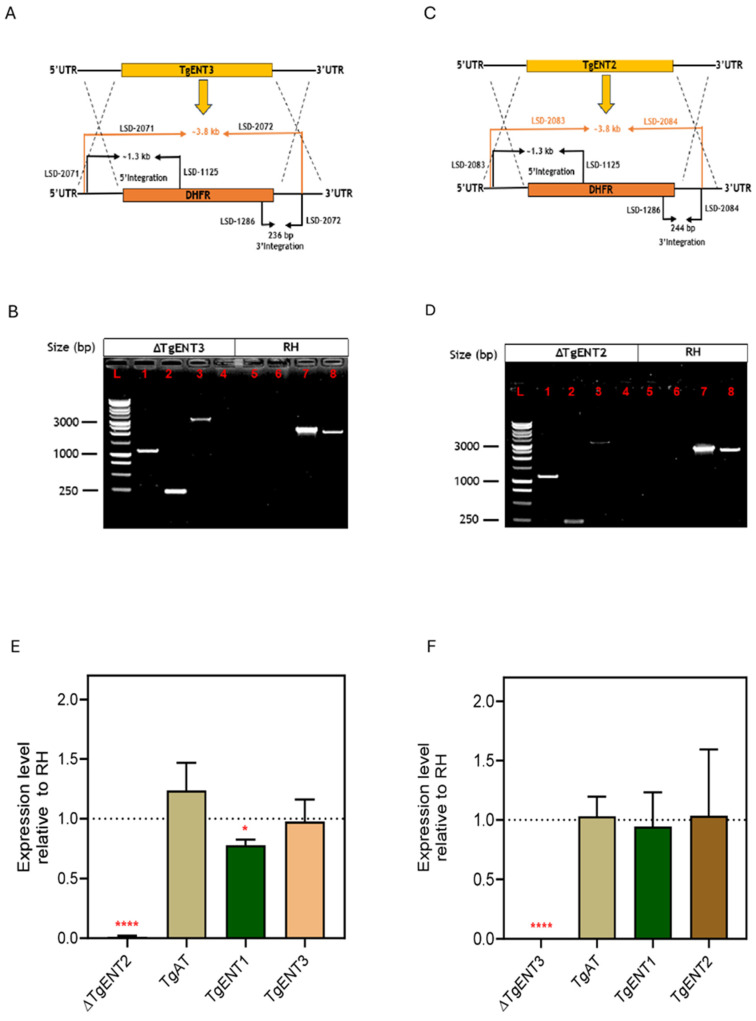
Verification of *TgENT3* and *TgENT2* knockout in RH cells. (**A**). Schematic depicting the genetic manipulation used to replace *TgENT3* with the DHFR cassette. Primers used in (**B**) are shown. (**B**). PCR confirmation of *TgENT3*-KO of DHFR integration after transfection of the RH cells. (**L**) 1 kb DNA Ladder; using the *TgENT3*-KO: (**1**) LSD-2071-(UTR-F of *TgENT3*) and LSD-1125-DHFR-R; ~1.3 kb; (**2**) LSD-1286 (DHFR-F) and LSD-2072 (UTR-R of *TgENT3*), 236 bp; (**3**) LSD-2071-(UTR-F of *TgENT3*) and LSD-2072-(UTR-R of *TgENT3*); ~3.8 kb; (**4**) LSD-2935 and LSD2936 (open reading frame primers of *TgENT3*); no band. Negative control, RH line: (**5**) LSD-2071-(UTR-F of *TgENT3*) and LSD-1125-DHFR-R; no band; (**6**) LSD-2072-(UTR-R of *TgENT3* and LSD-1286-DHFR-F; no band (**7**) LSD-2071 (UTR-F of *TgENT3*) and LSD-2072 (UTR-R of *TgENT3*); 2.2 kb); (**8**) LSD-2935 and LSD-2936 ~2 kb. (**C**). Schematic depicting the genetic manipulation used to replace *TgENT2* with the DHFR cassette. (**D**). PCR confirmation of *TgENT2*-KO in RH cell line after transfection of the RH cells. (**L**) 1 kb DNA Ladder (Promega); (**1**) LSD-2083 (UTR-F of *TgENT2*) and LSD-1125 (DHFR-R), ~1.3 kb; (**2**) LSD-2084 (UTR-R of *TgENT2*) and LSD-1286 (DHFR-F), 244 bp; (**3**) LSD-2083 (UTR-F of *TgENT2* and LSD-2084 (UTR-R- of *TgENT2*), ~3.8 kb; (**4**) LSD-2939 and LSD-2940 (ORF primers of *TgENT2*), no band. RH cell line as negative control: (**5**) LSD-2083 (UTR-F of *TgENT2*) and LSD-1125 (DHFR-R); (**6**) LSD-2084 (UTR-R of *TgENT2*) and LSD-1286 (DHFR-F); no band (**7**) LSD-2083 (UTR-F of *TgENT2*) and LSD-2084 (UTR-R of *TgENT2*), ~2.7 kb; (**8**) LSD-2939-F and LSD-2940-R, ~2.5 kb. (**E**). qRT-PCR analysis of *TgENTs* gene expression in the TgENT2-KO cell line. (**F**). qRT-PCR analysis of *TgENT*s gene expression in the TgENT3-KO cell line. *, *p* < 0.05; ****, *p* < 0.0001.

*TgENT2*-KO and *TgENT3*-KO were generated using CRISPR/Cas9 by transfecting RH parasites with the cassette amplified from the pDT7S4 template [28] and with the Tub-Cas9-YFP-pU6-ccdB-tracrRNA plasmid [35] containing the specific sgRNAs to cut the gene near the start or stop codon, respectively, together facilitating the replacement of the gene of interest (GoI) with the DHFR gene that confers pyrimethamine resistance for selection (Figure 1A,C). Correct integration was confirmed by PCR showing the presence of the ~3.8 kb DHFR amplicon and the absence of the targeted GoI in the transfectants (Figure 1B,D).

The absence of each targeted GoI was further confirmed at the mRNA level using qRT-PCR; gene expression was normalized to the catalase gene expression (Figure 1E,F). In both knockout strains, mRNA of the targeted gene could not be detected, but the mRNA level of the other three *TgENTs* was unchanged relative to the parental RH cells (except a minor, just-significant (*p* < 0.05, *t*-test) reduction in *TgENT1* mRNA in the *TgENT2*-KO), suggesting that there was no compensatory upregulation of other transporters after the deletion of either *TgENT2* or *TgENT3*, at least in culture and at the mRNA level.

#### 3.1.3. Creation of a *TgENT2/3* Double Knockout Cell Line (*ENT2/3*dKO)

**Figure 2:**
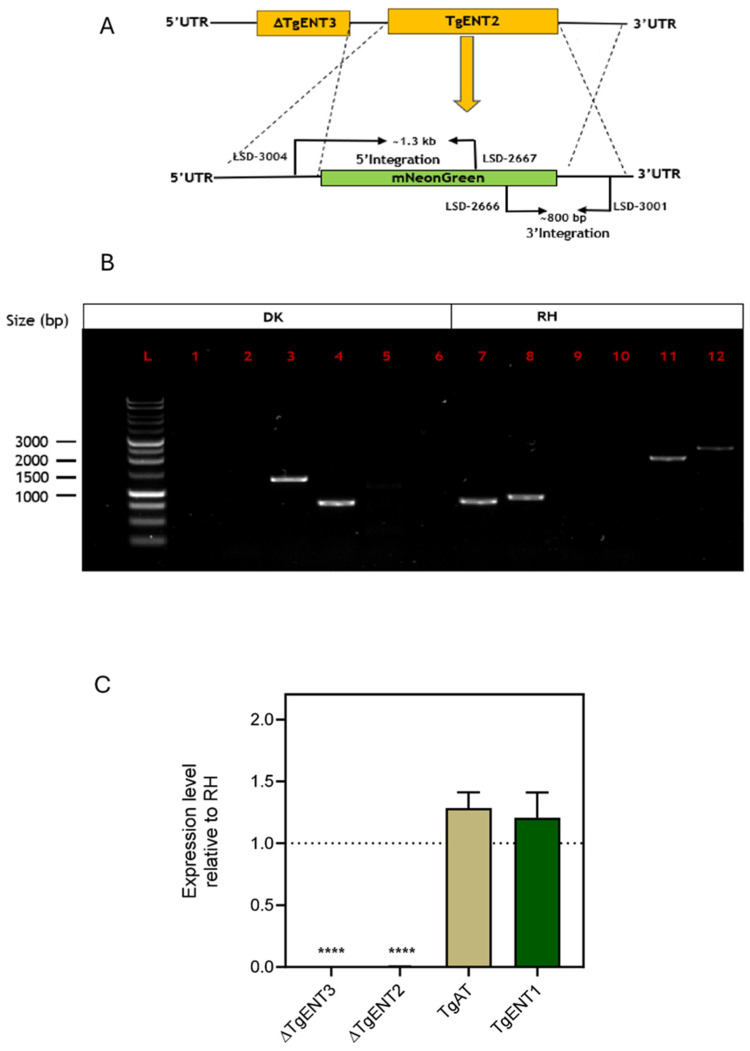
Construction and verification of *TgENT2/3*dKO in RH cells. (**A**). Schematic for the strategy of making a double knockout of *TgENT2* and *TgENT3*, indicating primer positions. (**B**). PCR confirmation of the *TgENT2/3*dKO in RH tachyzoites. (**L**) 1 kb DNA Ladder (Promega); (**1**) LSD-3004-F (5′ UTR of *TgENT2*) and LSD-3003-R (downstream start of *TgENT2*), no band; (**2**) LSD-3002-F (ORF of *TgENT2*) and LSD-3001-R (3′ UTR-R of *TgENT2*), no band; (**3**) LSD-3004 (5′ UTR of *TgENT2*) and LSD-2667 (mNG reverse), ~1.3 kb; (**4**) LSD-2666-F (mNG forward) and LSD-3001-R (3′ UTR-R of *TgENT2*), ~800 bp; (**5**) LSD-2935-F and LSD-2936-R (ORF of *TgENT2*), no band; (**6**) LSD-2939-F and LSD-2940-R (ORF of *TgENT3*), no band. RH cell line as a negative control: (**7**) LSD-3004 (5′ UTR of *TgENT2*) and LSD-3003-R (downstream start of *TgENT2*), ~750 bp; (**8**) LSD-3002-F (ORF of *TgENT2*) and LSD-3001-R (3′ UTR-R of *TgENT2*), 900 bp; (**9**) LSD-3004-F (5′ UTR of *TgENT2*) and LSD-2667-R (mNG ORF reverse); (**10**) LSD-2666-F (mNG ORF forward) and LSD-3001 (3′ UTR-R of *TgENT2*), ~800 bp; (**11**) LSD-2935-F and LSD-2936-R (ORF of *TgENT2*), ~2 kb; (**12**) LSD-2939-F and LSD-2940-R (ORF of *TgENT3*), ~2.5 kb. (**C**). qRT-PCR assessment of mRNA levels in TgENT2/3dKO. ****, *p* < 0.0001.

A *TgENT2/3*dKO strain was generated using *TgENT3*-KO as the parental line; the mNeonGreen (mNG) cassette [36] was used as a selectable marker for this construct (Figure 2A). A cassette whereby mNG is flanked by the 5′- and 3′-UTRs of *TgENT2* was amplified and transfected into *TgENT3*-KO cells. Transfectants expressing mNG were isolated using flow cytometry. Successful integration in the *TgENT2* locus was confirmed by PCR reactions (Figure 2B), and the absence of the *TgENT2* and *TgENT3* corresponding mRNAs was confirmed by qRT-PCR (Figure 2C). As in the single gene deletions, there was no significant upregulation of the two remaining *TgENT*s relative to their expression level in RH control cells.

#### 3.1.4. Deletion of *TgAT1* in the RH Cell Line

**Figure 3:**
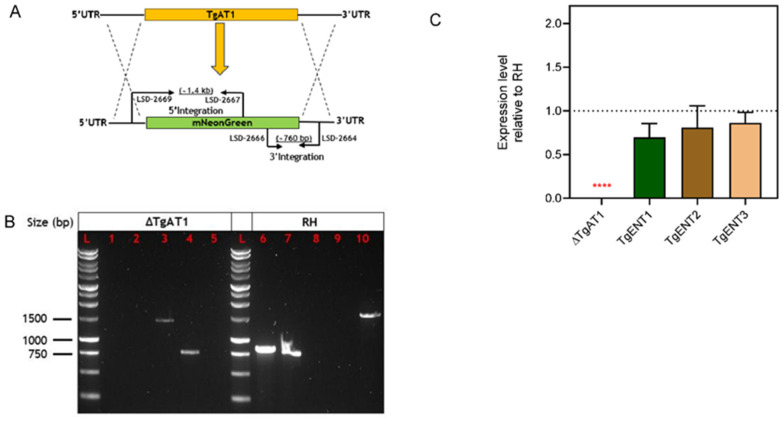
Construction and verification of *TgAT1*-KO in RH cells. (**A**). Schematic representing the genetic manipulation used to replace *TgAT1* with the mNeonGreen cassette. Primers used in (**B**) are shown. (**B**). PCR confirmation of *TgAT1*-KO using primers of *TgAT1* and mNeonGreen cassette; (**L**) 1 kb DNA Ladder (Promega); (**1**) LSD-2669-F (upstream of the start codon of UTR of *TgAT1*) and LSD-2668-R (downstream start codon of *TgAT1*), no band; (**2**) LSD-2665-F (upstream of the stop codon of *TgAT1*) and LSD-2664-R (downstream of stop UTR-R of *TgAT1*), no band; (**3**) LSD-2669-F (upstream of the start codon of UTR of *TgAT1*) and LSD-2667-R (mNeonGreen upstream reverse); band size (~1.4 kb); (**4**) LSD-2666-F (mNeonGreen downstream forward) and LSD-2664 (downstream of stop codon UTR-R of *TgAT1*); (~760 bp); (**5**) LSD-2937 and LSD-2938 (ORF of *TgAT1*), no band. RH cell line as a negative control: (**6**) LSD-2669-F (upstream of the start codon of UTR of *TgAT1*) and LSD-2668-R (downstream start codon of *TgAT1*); expected band (~860 bp); (**7**) LSD-2665-F (upstream of the stop codon of *TgAT1*) and LSD-2664-R (downstream of stop UTR-R of *TgAT1*); expected band (~796 bp); (**8**) LSD-2669-F (upstream of the start codon of UTR of *TgAT1*) and LSD-2667-R (mNeonGreen upstream reverse); no band expected in RH control; (**9**) LSD-2666-F (mNeonGreen downstream forward) and LSD-26664 (downstream of stop codon UTR-R of *TgAT1*); no band expected in RH control; (**10**) LSD-2937 + LSD-2938 (open reading frame of *TgAT1*); expected band (~1.5 kb). (**C**). qRT-PCR analysis of *TgENTs* gene expression in TgAT1-KO cells. ****, *p* < 0.0001.

The knockout of *TgAT1* by CRISPR-assisted homologous recombination and replacement with a DHFR cassette, as for *TgENT2* and *TgENT3*, was attempted multiple times but did not yield viable cells upon selection pressure with pyrimethamine, indicating that, at least in our RH strain and under the in vitro conditions used, *TgAT1* may be essential, unlike the other two *TgENT*s. We therefore created a new construct for replacement with mNG, flanked by the *TgAT1* UTRs (Figure 3A). Replacement with this cassette was followed by flow cytometry to isolate positive green fluorescent clones. Correct integration and *TgAT1*-KO were confirmed by PCR reactions (Figure 3B), and mRNA depletion was confirmed by qRT-PCR. Once again, the depletion of *TgAT1* had no significant effect on the mRNA levels of the other *TgENT* genes in support for the absence of a compensatory mechanism between those genes in *T. gondii* (Figure 3C).

#### 3.1.5. Creation of a Conditional Knockout of *TgENT1*

**Figure 4:**
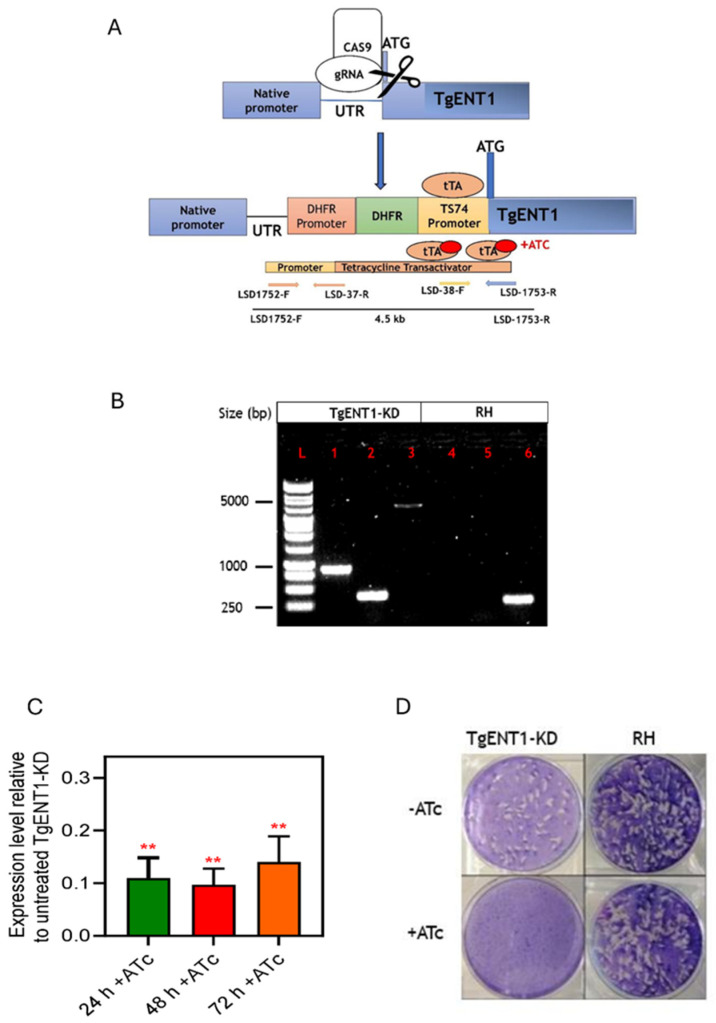
Construction and verification of the promotor replacement for *TgENT1*. (**A**). Schematic of the CRISPR/cas9-aided strategy for the introduction of the DHFR-TS74 construct upstream of the *TgENT1* start codon. (**B**). PCR validation of the promotor replacement for *TgENT1* in RH cells. (**L**) 1 kb DNA Ladder; (**1**) LSD-1752 (5′ UTR-F of *TgENT1*) and LSD-37 (DHFR ORF reverse), ~1 kb; (**2**) LSD-38-F (T7S4-F) and LSD-1753 (ORF-R of *TgENT1*), ~380; (**3**) LSD-1752 (5′ UTR of *TgNET1*) and LSD-1753 (ORF-R of *TgENT1*), ~4.5 kb; (**4**) LSD-1752 (5′ UTR-F of *TgENT1*) and LSD-37 (DHFR cassette reverse), no band; (**5**) LSD-38 (T7S4-F) and LSD-1753 (ORF-R of *TgENT1*), no band; (**6**) LSD-1752F and LSD-1753; band size ~280 bp. (**C**). qRT-PCR of *TgENT1* mRNA after addition of ATc, normalized to the expression of catalase. The amount of cDNA template used was 10 ng. The presented results are the mean and ± SEM of 3 independent biological replicates, each performed in triplicate. ** *p* < 0.01 by one sample *t*-test. (**D**). Plaque assay to assess in vitro growth of the *TgENT1*-KD strain (left) along with parental control (right) in HFF cells, in the presence and absence of ATc.

As mentioned, *TgENT1* is predicted to be essential to tachyzoites in vitro [47]. We therefore used promotor replacement (PR) to engineer a conditional depletion line for this gene under control of a tetracycline-inducible transactivator system, illustrated in Figure 4A. This technique relies on replacing the endogenous promotor with the tetracycline responsive promoter T7S4 [28,48]. Primers containing homology to 50 bp stretches of the 5′ UTR and the first exon promoted the insertion of the pyrimethamine-resistance (DHFR) cassette for selection followed by the T7S4 promoter that then controls transcription of the native copy of *TgENT1* (Figure 4A) (as described in Lacombe et al. [49]). In this system, the addition of anhydrous tetracycline (ATc) binds to the transactivator and prevents transcription, resulting in gene depletion. The resulting manipulation was verified by PCR and after transfection clones with the correct integration of the construct were selected on pyrimethamine and verified by PCR (Figure 4B); the new cell line was named *TgENT1*-KD. Depletion of the corresponding mRNA was tested by qRT-PCR, which showed a strong and stable knockdown of mRNA levels as early as 24 h (Figure 4C). Using a plaque assay, we showed that the *TgENT1*-KD cells did not replicate in the presence of ATc, whereas the parental RH cells did (Figure 4D), confirming that *TgENT1* is essential for growth in culture.

#### 3.1.6. Expression of *TgENTs* in a Leishmania Mexicana Cell Line Deficient in Nucleobase Transport

**Figure 5:**
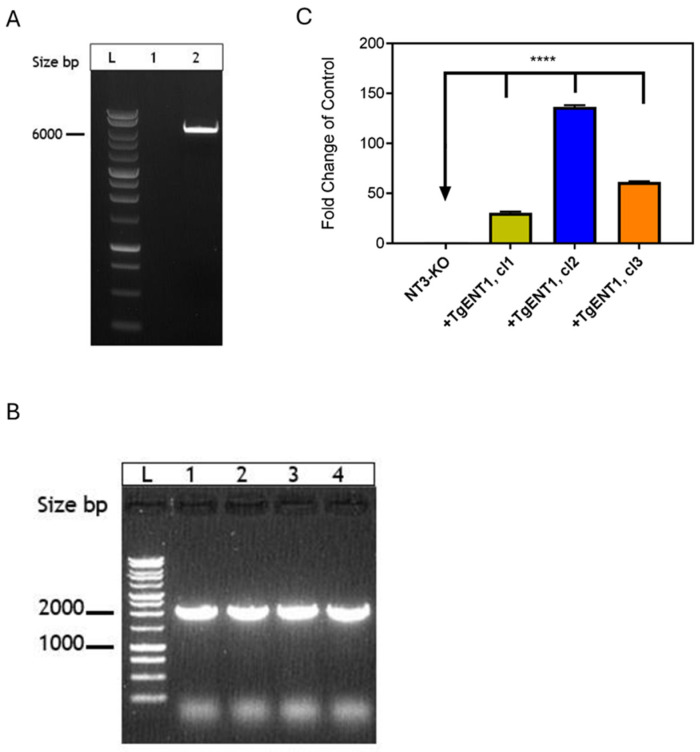
Construction of *Leishmania mexicana* cell lines expressing the *TgENT1* minigene. (**A**). pNUS-HcN vector digestion by *Nde*I and *Xho*I restriction enzymes run on 1% agarose gel; band size was 6.3 kb. (**L**) 1 kb DNA Ladder (Promega); (**1**): negative control water; (**2**): digested pNUS-HcN with the size band (6.3 kb). (**B**). PCR confirmation of the presence of *TgENT1* minigene in LmexNT3-KO clones grown out after transfection with pNUS/TgENT1 and selection on G-418; *TgENT1*-specifc forward primer HDK-1739 and reverse primer (3′ vector sequence) HDK-340. (**L**) 1 kb DNA Ladder (Promega); (**1**): clone 1; (**2**): clone 2; (**3**): clone3; (**4**): clone 4 (~2.2 kb). (**C**). The expression levels of *TgENT1* in Lmex-NT3-KO and compared to the control (*Lmex*NT3-KO) determined by qRT-PCR; expression levels in three independent clones are shown. ****, *p* < 0.0001 relative to the NT3-KO control (arrow).

We have previously shown the utility of expressing protozoan ENT-family transporters in *L. mexicana* cell lines that lack either nucleoside transport (ΔLmexNT1.1/1.2/2, “SUPKO”) or nucleobase transport (ΔLmexNT3, “LmexNT3-KO”) [31]. Here, we used that same procedure to express *TgENT1* in the *Lmex*NT3-KO strain. The *TgENT1* minigene was amplified by PCR from cDNA of the *T. gondii* RH strain and ligated into the *Nde*I and *Xho*I-digested plasmid pNUS-HcN [34] (Figure 5A). *TgENT1* was then ligated into the digested pNUS-HcN using the NEBuilder HiFi DNA Assembly Cloning Kit, creating plasmid pHDK295, and used to transform *E. coli*. Six positive clones were picked and the correct plasmid assembly verified by a diagnostic digestion with *Nde*I and *Xho*I and by PCR for *TgENT1* integration into pHDK295, followed by Sanger sequencing of the amplicon. Upon confirmation of the correct sequence, pHDK295 was transfected into *Lmex*NT3-KO and selected on 50 µg/mL of G-418. Clones were obtained by limiting dilution, and the presence of the plasmid in these clones was confirmed by PCR (Figure 5B). The relative expression level of *TgENT1* in these clones was assessed by qRT-PCR, showing highest expression in clone 2 (Figure 5C), which was therefore used for further analysis of the transporter function (see below).

### 3.2. Investigation into the Physiological Role of the TgENT Transporters

#### 3.2.1. Is *TgENT2* or *TgENT3* Involved in Pyrimidine Uptake in *T. gondii*?

Messina et al. [26] reported that TgAT1 and TgENT3 gene products are important in bradyzoite formation and thus for persistence of *T. gondii* in the host. Several authors have also demonstrated that *TgENT1* is essential in tachyzoites [26,46,47]. However, the role of the individual transport proteins in *T. gondii* physiology is poorly defined. We have recently reported the identification of a uracil/uridine transporter, TgUUT, in *T. gondii* tachyzoites [13] that is potentially important for anti-toxoplasmic chemotherapy development. Our starting point was therefore to try to establish which of the four *TgENT* genes encodes this pyrimidine transporter, using the knockout and knockdown lines we generated.

**Figure 6:**
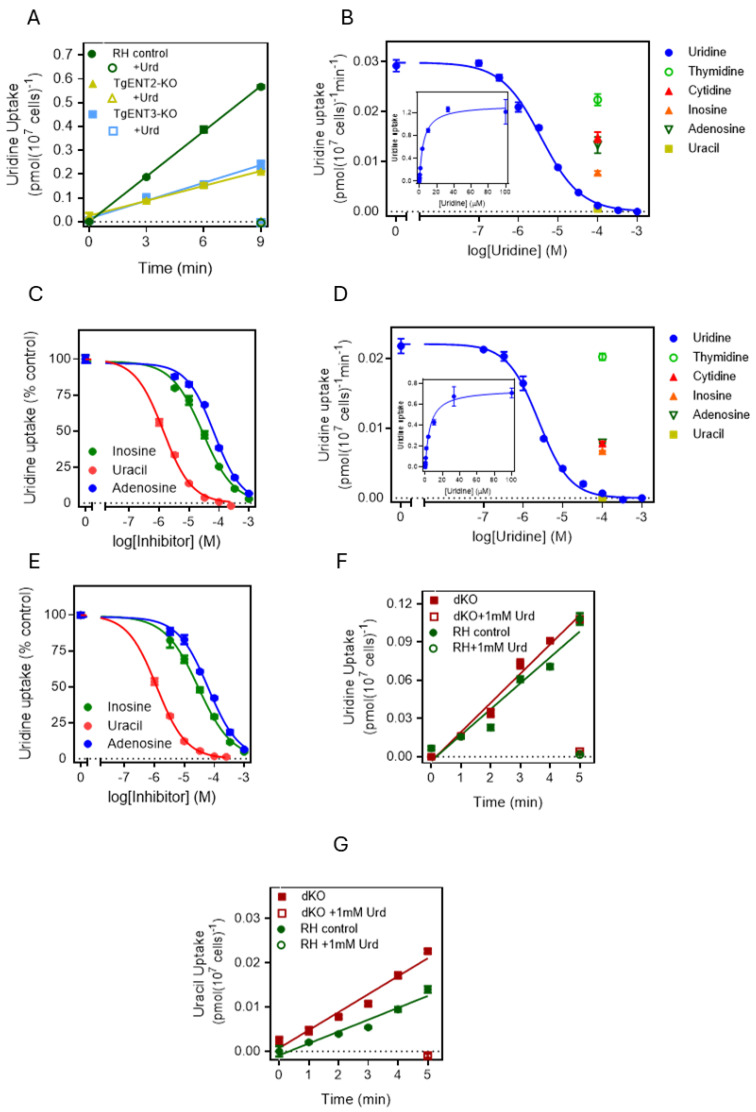
Pyrimidine transport in tachyzoites of the *TgENT2* and *TgENT3* knockout strains. (**A**). Time course of 0.1 µM [^3^H]-uridine transport by the indicated strains in the absence (0, 3, 6 and 9 min) or presence (9 min) of 1 mM unlabeled uridine. (**B**,**D**). Dose–response inhibition of unlabeled uridine or of 100 µM of other purines and pyrimidines of 0.1 µM [^3^H]-uridine transport by TgENT2-KO and TgENT3-KO cells, respectively. *Inset*: conversion of the uridine inhibition data to a Michaelis–Menten saturation curve. (**C**,**E**). Dose-dependent inhibition of 0.1 µM [^3^H]-uridine transport by TgENT2-KO and TgENT3-KO cells by uracil, respectively, and inosine and adenosine, expressed as % of uninhibited control. (**F**). Time course of 0.1 µM [^3^H]-uridine transport by TgENT2/3dKO and RH tachyzoites. (**G**). The same as frame F but 0.1 µM [^3^H]-uracil transport.

As the TgAT1 gene product has already been characterized as a purine-specific transporter [24,25], and *TgENT1* is essential and thus harder to manipulate, we first performed uridine transport assays with the knockout strains of *TgENT2* and *TgENT3* in parallel with the parental strain as a control. Figure 6A shows that the two knockout strains displayed identical rates of uridine uptake, although both showed an apparently lower rate than the control, requiring us to investigate uridine uptake in both strains in detail.

The knockout strain *TgENT2*-KO displayed robust uptake of 0.1 µM [^3^H]-uridine, with an average K_m_ of 5.29 ± 0.58 µM and a V_max_ of 0.025 ± 0.002 pmol (10^7^ cells)^−1^min^−1^ (Figure 6B), which is not significantly different from the corresponding values in the parental cell line (Table 2). Indeed, the inhibition constants for uracil, inosine and adenosine were also highly similar to the parental line (Figure 6C, Table 2; *p* > 0.05). The same pattern was observed with the *TgENT3*-KO (Figure 6D,E), and Table 2 lists the three sets of parameters side by side. These data allowed for two possible hypotheses: (a) neither gene encodes the TgUUT1 uridine/uracil transporter, or (b) they both express a highly similar uridine/uracil transporter and only the knockout of both genes simultaneously would create a cell line that was deficient in the uptake of these pyrimidines. The *TgENT2/3*dKO strain allowed us to distinguish between these possibilities. Figure 6F shows that uridine uptake was identical in *TgENT2/3*dKO and parental cells, and Figure 6G shows that uracil uptake was somewhat higher in the dKO cells, certainly not lower. Overall, these data show that neither *TgENT2* nor *TgENT3* encode the TgUUT1 pyrimidine transport activity.

#### 3.2.2. Investigating the Role of *TgENT1* in Pyrimidine Transport

As the *TgENT1* gene could not be deleted, its role in pyrimidine salvage by tachyzoites was investigated using the promotor replacement line, with expression suppressed upon addition of ATc. As the level of mRNA depletion was almost maximal at 24 h (Figure 4D) and there is a risk of secondary effects on other cellular processes the longer the essential gene is suppressed, transport was assessed at 24 h and at 48 h, and the rates in the presence and absence of ATc were compared.

**Figure 7:**
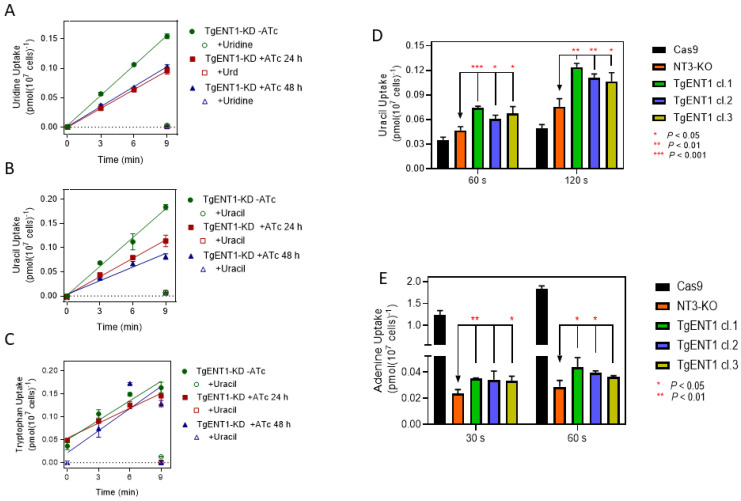
*TgENT1* is a pyrimidine transporter. (**A**–**C**). Transport of 0.1 µM [^3^H]-uridine, 0.1 µM of [^3^H]-uracil or [^3^H]-tryptophan, respectively, by *T. gondii* tachyzoites engineered with promotor replacement for the suppression of TgENT1 expression in the presence of anhydrous tetracycline (ATc). Cells were cultured in the presence of ATc for either 24 h or 48 h prior to the uptake experiment and then compared to cells cultured in the absence of ATc (green circles); for each category, uptake was also measured at 9 min of incubation in the presence of 1 mM unlabeled substrate (uridine, Urd). (**D**). Uptake of 0.1 µM of [^3^H]-uracil by *L. mexicana* NT3-KO promastigotes expressing the *TgENT1* minigene. Cas9 is the parental strain of NT3-KO, i.e., it does express the LmexNT3 nucleobase transporter. Three independent clones of NT3/TgENT1 were assessed, each in triplicate. (**E**). The same as frame D but with 0.1 µM [^3^H]-adenine.

The uptake of 0.1 µM [^3^H]-uridine was highly significantly reduced in *TgENT1*-KD cells incubated for either 24 h or 48 h with ATc, to 62.7% of control (*p* < 0.0001, F-test) or 66.5% of control (*p* = 0.0004), respectively; the linear regression slopes of the 24 h and 48 h knockdown lines were not significantly different from each other (*p* > 0.05) (Figure 7A). Similarly, uptake of 0.1 µM [^3^H]-uracil was significantly lower after incubation with ATc for 24 h (63.8% of control, *p* = 0.0063) or 48 h (47.4% of control, *p* = 0.0048) (Figure 7B). As a control, we also conducted the same experiment with 0.1 µM [^3^H]-tryptophan, and in this case the incubation with ATc for either 24 h or 48 h did not affect the rate of uptake (Figure 7C). We thus conclude that the suppression of *TgENT1* mRNA production specifically diminishes the transport of uridine and uracil in tachyzoites.

For further verification, we next expressed *TgENT1* in our recently described *Leishmania mexicana* NT3-KO strain, which displays a *null* background for purine nucleobase transport and a very low background for uracil uptake, exactly as described for the *Trypanosoma vivax* NT3 nucleobase transporter [31] and the *T. cruzi* NB1 adenine transporter [33]. Expressed from an episome in this system, the TgENT1 gene product was able to significantly increase 0.1 µM [^3^H]-uracil transport over the background, in a time-dependent fashion, in all three independently generated clones expressing the transporter (Figure 7D). As a further control, we assessed the uptake of [^3^H]-adenine in the same *TgENT1*-expressing clones, for which the *Lmex*NT3-KO cells give an exceptionally sensitive system, as *L. mexicana* adenine uptake in those cells was only 1.9% and 1.6% of control at 30 s and 60 s incubation time, respectively, in the *L. mexicana*-Cas9 T7 strain [29] that is the parent line of *Lmex*NT3-KO [31] (Figure 7E). [^3^H]-adenine was just detectable and very slightly elevated in two out of the three TgENT1-expressing clones tested at each timepoint but remained <3% of the rate in cas9 cells (Figure 7E). However, the data indicate that *TgENT1* has a much higher capacity for uracil uptake than for adenine uptake. The combined data with the *TgENT1*-KD tachyzoites and the expression of *TgENT1* in *L. mexicana* NT3-KO cells strongly suggest that *TgENT1* encodes TgUUT1.

### 3.3. Effect of Transporter Knockouts on Sensitivity to 5-Fluorinated Pyrimidines

If the above conclusion, that *TgENT1* is likely to be the gene encoding the TgUUT1 uracil/uridine uptake activity, is correct, sensitivity to pyrimidine antimetabolites such as 5F-uracil, 5F-uridine and 5F-2′-deoxyuridine, all potent agents against *T. gondii* in vitro [13], should not vary with the expression levels of the other three TgENTs.

All three pyrimidine analogues that were screened in our previous study [13] showed good activity against *T. gondii* RH with a cytostatic effect at lower concentrations and a cytocidal effect at high concentrations against HFF monolayer cells. The best activity was displayed by 5-fluorouridine, with an EC_50_ of 0.45 µM, making it 27.6-fold more potent than the standard drug sulfadiazine (Table 3).

The pyrimidine analogues displayed essentially the same activity against the knockout strains of *TgENT2*, *TgENT3* and *TgAT1*, with the sole exception of a just-significant sensitization by the *TgENT2*-KO strain to 5F-uridine (EC_50_ = 0.33 µM; *p* = 0.02) and with a similar trend in *TgENT3* cells that did not reach significance (EC_50_ = 0.36 µM; *p* = 0.07). In the double knockout strain that EC_50_ value further decreased to 0.29 µM (*p* = 0.003), and the values for 5F-uracil and 5F-2′-deoxyuridine were also significantly lower. This trend could be due to a possible upregulation in the pyrimidine salvage pathways or perhaps the internal distribution of pyrimidine metabolites in the cell. The observations with the 5F-pyrimidine antimetabolites are fully consistent with *TgAT1*, *TgENT2* and *TgENT3* having no significant role in uracil and uridine transport. Unfortunately, it was not possible to test 5F-pyrimidine sensitivity on a *TgENT1*-KO, which we could not isolate as it is lethal, nor with the knockdown line which shows an early growth arrest, as the assay requires a healthy, growing cell population.

## 4. Discussion

In this study, we aimed to identify the gene encoding the *T. gondii* uracil/uridine transporter TgUUT1 that we recently described in isolated tachyzoites [13]. Tachyzoites with a disrupted pyrimidine biosynthesis pathway (i.e., auxotrophs) are able to grow in vitro on uracil or uridine as the sole pyrimidine source [12] but not on thymidine, as the species lack thymidine kinase [50] and is unable to take up [^3^H]-thymidine [13]. The lack of thymidine salvage makes *Toxoplasma*, like *Plasmodium* species, particularly sensitive to antifolates, which are the first-line treatment for toxoplasmosis, as the parasite relies completely on the synthesis of thymidine from 2′-deoxyuridine by folate-dependent thymidylate synthase [12]. The lack of thymidine kinase similarly underpins the remarkable sensitivity to 5F-uracil, 5F-uridine and 5F-2′-deoxyuridine as their mode of action, as the metabolite 5F-2′-deoxyuridine monophosphate (5F-dUMP) inhibits thymidylate synthase, and 5F-2′dUTP is incorporated in place of TTP into nucleic acids, leading to growth arrest and fatal DNA damage [51]. The reliance on thymidylate synthase is shared with the related *Plasmodium* species, as these are also unable to take up thymidine [44], yet *Plasmodium* species are very much less sensitive to 5F-uracil, 5F-uridine and 5F-2′-deoxyuridine but highly sensitive to 5F-orotate [52] as, unlike *T. gondii* [13], orotate is the only pyrimidine they are able to take up or incorporate into their nucleic acids [53,54]. This provides a demonstration for the importance of transporters in the effectiveness of fluorinated pyrimidines and other antimetabolites.

In agreement with other recent reports [26,46], we identified four potential nucleoside transporter genes in the *Toxoplasma* genomes available on toxoDB.org, all of the ENT family. Of these, we have previously characterized *TgAT1* in great detail and shown that it encodes a high-affinity purine transporter with no measurable affinity for uracil and uridine [25]. As TgUUT1 displays high affinity for both uracil and uridine and low affinity at best for purine nucleosides [13], TgAT1 does not encode the uracil/uridine transporter. In the current study, we made gene deletion strains for *TgENT2* and *TgENT3* as well as the double knockout *TgENT2/3*dKO and showed that these deletions did not materially change [^3^H]-uridine uptake parameters or reduce sensitivity to 5F-pyrimidines, leading us to conclude that neither of these genes encode the TgUUT1 activity either, leaving *TgENT1* as the most likely candidate.

As the gene could not be deleted [26], we created a cell line where its expression is controlled by the tetracycline responsive promoter T7S4 and thus suppressed in the presence of ATc. As expected, the abundance of the corresponding mRNA was reduced by >80% at 24 h, 48 h and 72 h. In order to minimize any secondary effects from the knockdown, we assessed the uptake of 0.1 µM [^3^H]-uridine or 0.1 µM uracil at 24 h and 48 h and compared the rate in the presence and absence of ATc. The uptake rate for both substrates, and at both timepoints, was highly and significantly reduced, while the rate of uptake of 0.1 µM [^3^H]-tryptophan was unaffected. Moreover, the expression of *TgENT1* in a *Leishmania* cell line with a low endogenous rate of uracil uptake increased the uptake of 0.1 µM [^3^H]-uracil significantly in all three independent clones assayed while barely affecting [^3^H]-adenine uptake rates despite the near-zero uptake level for the purine nucleobase.

The combined data presented strongly suggest that *TgENT1* encodes the TgUUT1 uracil/uridine transport activity. Although the uracil and uridine uptake was not completely abolished upon *TgENT1* knockdown, this could theoretically reflect a small contribution from low-affinity uptake through a secondary transporter. However, we have conclusively shown, in this and our previous paper [13], that none of the other TgENTs make a significant contribution to uracil or uridine uptake at low permeant concentrations. The notion of uracil and uridine uptake being mediated by a single transporter is further strongly supported by the data presented in [13] that uptake of both uracil and uridine is reciprocally and fully inhibited by both high-affinity substrates, 5-FU, and even low-affinity inhibitor inosine, in a monophasic way with a Hill slope of approximately −1. These are all indicators of a single transporter being responsible for the uptake of uracil and uridine.

Logically, however, that leaves the possibility of a non-ENT family transporter also being involved in this process. Such a hypothetical transporter is unlikely to be of the CNT family either nor of any of the other known nucleobase or nucleoside transporter families [14,55] as, to the best of our knowledge, no members of any of these gene families have to date been identified in protozoan genomes. However, it should be noted here that the genes encoding the high-affinity uracil transporters of *Leishmania* [56] and *Trypanosoma* species [57,58] have not yet been identified, and we have speculated that they may be encoded by a different, as yet unknown transporter family [59]. However, these kinetoplastid transporters are extremely selective for uracil only, whereas TgUUT1 displays identical affinity for uracil and uridine [13]. Substantial efforts from our group [60,61] and the group of Marc Ouellette [62] have not identified any such novel uracil transporter family in kinetoplastids.

We were not able to verify that knockdown of TgENT1 leads to resistance to the 5-fluoropyrimidines, because the drug sensitivity assay requires dividing cells and gene depletion leads to immediate growth arrest. Moreover, 5-FU and its analogues act on nucleic acids synthesis and stability [51]; thus, testing on cells already in growth arrest from the TgENT1 knockdown will doubtless yield a different EC_50_ from the no-ATc (non-induced) control, but this cannot then be simply attributed to reduced uptake rates. The small increase in pyrimidine transport in the *L. mexicana* NT3-KO + TgENT1 cells was also not likely to cause a significant increase in sensitivity to the fluorinated pyrimidines in an already 5-FU-sensitive background, as *Leishmania* express an efficient uracil/5-FU carrier [56]. Furthermore, overexpression of *TgENT1* in *T. gondii* tachyzoites is not expected to change the EC_50_ values of the fluoropyrimidines. TgUUT1 displays very high affinity for the carrier, similar to that of uracil and uridine [13], and it is more likely that the rate-determining step is metabolic, in the conversion of 5-FU or 5-FUrd to the (2′-deoxy)uridine triphosphate and incorporation into nucleic acid. We must therefore infer from our previous data [13] that TgENT1 is highly likely also responsible for the uptake of 5-fluoropyrimidines, although we have not shown this directly in the current manuscript.

In conclusion, we believe that *TgENT1* encodes the *T. gondii* uracil/uridine transporter TgUUT1, responsible for the uptake of 5F-pyrimidines with high activity against this parasite. Since the TgENT1 transport activity is essential, as is the thymidine synthase pathway that mediates the action of these compounds, its use would not be expected to lead to a rapid onset of resistance.

## Figures and Tables

**Table 2:** Kinetic parameters of uridine transport in three cell lines.

	TgENT2-KO		TgENT3-KO		RH ^1^	
	Exp. Value	*p* Value	Exp. Value	*p* Value	Exp. Value	
K_m_	5.29 ± 0.58	>0.05	3.78 ± 0.92	>0.05	3.34 ± 0.82	µM
V_max_	0.025 ± 0.002	>0.05	0.011 ± 0.002	>0.05	0.020 ± 0.004	pmol (10^7^ cells)^−1^min^−1^
Uracil K_i_	0.96 ± 0.17	>0.05	1.06 ± 0.14	>0.05	1.15 ± 0.07	µM
Inosine K_i_	32.2 ± 3.3	>0.05	37.7 ± 3.82	>0.05	28.2 ± 4.1	µM
Adenosine K_i_	77.4 ± 10.7	>0.05	58.9 ± 1.10	0.002	111 ± 6	µM

All values are average ± SEM for *n* ≥ 3. Columns show either the experimental value obtained (Exp. value; *n* ≥ 3) or the *p* value relative to the RH control using an unpaired two-tailed *t*-test. ^1^ Values for the control RH cell line were taken from [13] for comparison.

**Table 3:** In vitro drug screening and HFF cytotoxicity using pyrimidine nucleobase and nucleoside analogous on different cell lines of *T. gondii* RH intracellular tachyzoites, TgAT1-KO, TgENT1-KO, TgENT2-KO and TgENT2/3dKO.

ID	RH	TgAT1-KO		TgENT2-KO		TgENT3-KO		TgENT2/3dKO		HFF Cytotoxicity		
	EC_50_ ± SEM ^a^	EC_50_ ± SEM	*p* ^b^	EC_50_ ± SEM	*p* ^b^	EC_50_ ± SEM	*p* ^b^	EC_50_ ± SEM	*p* ^b^	EC_50_ Cytostatic ^a^	EC_50_ Cytocidal ^a^	SI
**5-FU**	1.13 ± 0.16	0.70 ± 0.21	0.17	1.36 ± 0.26	0.49	0.82 ± 0.24	0.34	0.51 ± 0.08	0.03	2.49 ± 0.43	3074 ±339	2720
**5-FUrd**	0.45 ± 0.02	0.89 ± 0.42	0.35	0.33 ± 0.02	0.02	0.36 ± 0.03	0.07	0.29 ± 0.002	0.003	0.11 ± 0.10	20.4 ± 1	45
**5-F-2′dUrd**	0.67 ± 0.07	0.41 ± 0.14	0.16	0.84 ± 0.07	0.14	0.56 ± 0.07	0.28	0.40 ± 0.06	0.04	0.19 ± 0.05	1478 ± 512	2205
**Sulfadiazine**	12.4 ± 1.08	3.95 ± 0.29	0.0003	7.15 ± 0.19	0.01	8.12 ± 0.22	0.02	5.74 ± 0.29	0.004	--	--	--
**PAO**	--	--	--	--	--					--	0.06 ± 0.003	

All EC_50_ values are averages and SEM of at least three independent experiments, given in µM. SI, selectivity index (HFF cytocidal EC_50_/RH EC_50_). PAO. ^a^ Values taken from [13]. ^b^ *p* value is based on an unpaired Student *t*-test (RH EC_50_ versus knockout *TgENT* gene EC_50_).

## Data Availability

All relevant data are contained in this manuscript and the Supplementary Materials.

## Supplementary Materials

The following supporting information can be downloaded at: https://www.mdpi.com/article/10.3390/pathogens15030266/s1, Table S1: PCR master mix for Phusion High-Fidelity DNA Polymerase; Table S2: The PCR condition for Phusion High-Fidelity DNA Polymerase; Table S3: Primers for TgENT1 ligation into pNUS-HcN and post-transfection integration confirmation in *L. mex* NT3-KO; Table S4: sgRNA primers for generating knockouts (*TgENT2*, *TgENT3*, *TgAT1*, knockdown of *TgENT1*, and DK in *TgENT3*) in *T. gondii*; Table S5: List of primers designed and used for generation and confirmation of TgAT1-KO, TgENT1, TgENT2, TgENT3 and DK (ΔTgENT2/3); Table S6: Platinum SuperFI™ Green PCR Master Mix; Table S7: PCR (SuperFi™) program; Table S8: GoTaq G2 Hot Start Green Master Mix DNA Polymerase; Table S9: PCR conditions for GoTaq DNA Polymerase; Table S10: List of primers designed and used in qRT-PCR.

## Abbreviations

The following abbreviations are used in this manuscript:

ENT	Equilibrative Nucleoside Transporter
TgUUT1	*Toxoplasma gondii* Uracil/Uridine Transporter 1
TgAT1	*Toxoplasma gondii* Adenosine Transporter 1
