# Supplementary material for: Identification of TgENT1 as the TgUUT1 Uracil/Uridine Transporter of Toxoplasma gondii
Source: Pathogens. 2026 Mar 2;15(3):266. doi: 10.3390/pathogens15030266 (PMC13029480; doi:10.3390/pathogens15030266)
Supplement: Supplementary file 1 [file pathogens-15-00266-s001.zip › pathogens-4149024-supplementary.pdf]

Identification of TgENT1 as the TgUUT1 uracil/uridine transporter of *Toxoplasma gondii*.

Hamza A.A. Elati[1,2], Mariana Ferreira Silva[1], Lilach Sheiner[1,a*], and Harry P. De Koning[1,a,*]

1. School of Infection and Immunity, College of Medical, Veterinary and Life Sciences, Glasgow Centre for Parasitology, University of Glasgow, Glasgow G12 8TA, United Kingdom
2. Department of Pharmacology and Toxicology, Pharmacy College, University of Elmergib, Al Khums, Libya.

*, Corresponding authors. Email lilach.sheiner@glasgow.ac.uk ; harry.de-koning@glasgow.ac.uk; tel. +44 - 141 3303753

[a], these authors contributed equally

Table S1: PCR master mix for Phusion High-Fidelity DNA Polymerase.

No.	Component	Volume
1	dd H_2O	34 µL
2	5x Phusion GC buffer	10 µL
3	10 mM dNTP	1 µL
4	10 µM Forward primer	2 µL
5	10 µM Reverse primer	2 µL
6	Genomic DNA or cDNA	0.5 µL
7	Phusion polymerase	0.5 µL
8	Total of reaction	50 µL

TableS2: The PCR condition for Phusion High-Fidelity DNA Polymerase.

**Hot lid on 110°C**		**Cycle No**
Initial denaturation	98°C for 30 s	
Denaturation	98°C for 10 s	X35
Annealing	*°C for 40 s (*: Best melting temperature based on a gradient PCR)	

Extension	72°C for * s (*:30 s /kb of DNA of interest)	
Polymerisation	72°C for 10 min	
Final	Store at 10°C - ∞	Hold

Table S3: Primers for TgENT1 ligation into pNUS-HcN and post-transfection integration confirmation in *L. mex* NT3-KO.

Primer name	Position	Restriction site	Sequence	Amplicon name
HDK-1739	F	*NdeI*	CTTCCACTTGTCAAGCGAATTCCATATGATGGCTGGCCTGGACACTTTGG	TgENT1 in pNUS-HcN
HDK-1740	R	*XhoI*	CTCAGTGGTGGTGGTGGTGGTGCTCGAGGTACGCTAAGGCTGAGTAGACACCTGC	
HDK-340	R	-	CGTGGAGCAGCTGAAGGACA	pNUS-HcN-R
M13	F	-	CAGGAAACAGCTATGAC	-

Table S4: sgRNA primers for generating knockouts (*TgENT2, TgENT3, TgAT1,* Knockdown of *TgENT1,* and DK in (*TgENT3*) in *T. gondii*.

Uppercase = 20-nucleotides sgRNA target; lowercase = Cas9 backbone overhang.

**No.**	**Primer name**	**Sequence (5’ 3’)**
LSD-2073	5' sgRNA primer (Δ*TgENT3*)	aagttAGATTCTTCCTCGAAATGTCg
LSD-2074	3' sgRNA primer (Δ*TgENT3*)	aaaacGACATTTCGAGGAAGAATCTa
LSD-2085	5' sgRNA primer (TgENT2)	aagttGAGGGTAGGAAGGCTCTTCAg
LSD-2086	3' sgRNA primer (TgENT2)	aaaacTGAAGAGCCTTCCTACCCTCa
LSD-1748	5' sgRNA primer (TgENT1)	aagttAGTGCAGTCGCAAAAATGGCg
LSD-1749	3' sgRNA primer (TgENT1-KD)	aaaacGCCATTTTTGCGACTGCACTa
LSD-2919	5' sgRNA primer (TgAT1)	aagttGCTCATTTGCTGCGCCGCAAg
LSD-2920	3' sgRNA primer (TgAT1)	aaaacTTGCGGCGCAGCAAATGAGCa
LSD-2915	5' sgRNA primer (TgENT2-to make DK in ΔTgENT3	aagttGTAACCCGCAGCGCTCGTGAg
LSD-2916	3' sgRNA primer (TgENT2-to make DK in ΔTgENT3	aaaacTCACGAGCGCTGCGGGTTACa

Table S5: List of primers designed and used for generation and confirmation of TgAT1-KO, TgENT1, TgENT2, TgENT3 and DK (ΔTgENT2/3)

Name	Position	Sequence	Amplicon name
LSD-2937	F	ATGAGTACAATCGAAGAGAGAG	Open Reading Frame TgAT1
LSD-2938	R	CTAGTAAGCGAGAGCGAGGTA	Open Reading Frame TgAT1
LSD-3001	R	GAGATGTTGACCTCCTGGAG	Downstream of Stop (Reverse) UTR of TgENT2
LSD-3002	F	AAGCTTGTGCCGAACCTT	Upstream of Stop (Forward) of TgENT2
LSD-3003	R	GATAATCGATTCCGGGCCTTC	Downstream of Start (Reverse) of TgENT2
LSD-3004	F	GAACCTACACTGTGCTCTTGG	Upstream of Start (Forward) UTR of TgENT2
LSD-2664	R	gcgcacagatggtgaatcatc	Diagnostic UTR-R of TgAT1(Downstream of stop codon)
LSD-2665	F	CCGCACCTCTTGAACATTGGTC	Diagnostic: Upstream of Stop (Forward) of TgAT1
LSD-2666	F	gttccgtaagacggagctcaa	mNeonGreen Downstream Forward
LSD-2667	R	ctgcattgtgcgatggacttg	mNeonGreen Upstream Reverse
LSD-2668	R	AAGCAAAGCGAATTTGGTGCGG	Diagnostic: Downstream of Start (Reverse) of TgAT1

LSD-2669	F	GTGCGAGGTTCTGTTCTCCTT	Diagnostic UTR-F of TgAT1 (Upstream of the start codon
LSD-2069	F	TTTCCGTTGGTGGACTATCGATAGCGCGACACGAGA GATTCTTCCTCGAAaagcttcgccaggctgtaaatcc	5’UTR of TgENT3/DHFR cassette Flank
LSD-2070	R	CCTCCGCTAGTCCGGGCCTGGTCCTCTGAATTCGTT CGCTGTCACTCTCGggatcgatcccccggtttgc	3’UTR of TgENT3/DHFR cassette Flank
LSD-2071	F	TCGTTGTTTTGTCTCGGCGCG	5’UTR-TgENT3
LSD-2072	R	CACACGTTCCGCTCTCACCCT	3’UTR-TgENT3
LSD-2935	F	ATGTCCGGGTCAAAGTC	Open Reading Frame TgENT3
LSD-2936	R	CTAGTACGGTGCAGCGG	Open Reading Frame TgENT3
LSD-1286	F	gatgtgcgtgtatccactc	DHFR-Downstream Forward
LSD-1125	R	ggccgttgttgatgcc	DHFR-Upstream Reverse
LSD-2081	F	GGGGACGCAGTCGTCTGACGCCCATCAACTCTGGC GGATTCGGGCATACCaagcttcgccaggctgtaaatcc	5’UTR of TgENT2/DHFR cassette Flank
LSD-2082	R	CTGTCACCTGAGAGACAAACAGATGCCAGATTCGCA TGAGCCACTCTCATggatcgatcccccggtttgc	3’UTR of TgENT2/DHFR cassette Flank
LSD-2083	F	TGGTCATTGAGTGCACAGAG	5’UTR-TgENT2

LSD-2084	R	TCAGCACTCCACTGTAAACC	3’UTR-TgENT2
LSD-2939	F	ATGAAGAGCCTTCCTACCCT	Open reading frame TgENT2
LSD-2940	R	TTAGTACACCAGGAAAGCAA	Open reading frame TgENT2
LSD-2662	F	gtggacaggactacgatttgtttgatgtttctaacattttccctcgggggggcaagaatt	5’UTR of TgAT1/mNeonGreen Cassette Flank
LSD-2663	R	taaatccccttggcggtcgttctcggtctcattcgccaaaAAGCTTTTACATCCGTTGCC	3’UTR of TgAT1/mNeonGreen Cassette Flank
LSD-2913	F	CTGTCACCTGAGAGACAAACAGATGCCAGATTCGCATGAGCCACTCTCATccctcgggggggcaagaatt	5’UTR of TgAT2/mNeonGreen Cassette Flank to make DKO
LSD-2914	R	GGGGACGCAGTCGTCTGACGCCCATCAACTCTGGCGGATTCGGGCATACCaagcttttacatccgttgcc	3’UTR of TgENT2/mNeonGreen Cassette Flank to make DKO
LSD-1750	F	GATGACCGACGCTCGGTTTTCCCCCAGTCCATCCAAGTGCAGTCGCAAAAaagcttcgccaggctgtaaatcc	5’UTR of TgENT1/DHFR flank to make KD
LSD-1751	R	GGCCCGTCCAGGTCAACATCTTCGAGGGCCAAAGTGTCCAGGCCAGCCATtggttgaagacagacgaaagcagttg	TgENT1/T7S4 flank to make KD
LSD-1752	F	GCACGCTCGTATCTAGAGTC	PR Test-UTR-F of TgENT1
LSD-1753	R	AGAAAAGCAGCCTTGTATGCG	PR Test-of TgENT1

LSD-37	R	CACGGTTATCAAACCCGAG	DHFR-Cassette-R
LSD-38	F	CGGTTCGCTTGAAGAAGG	T7S4-Cassette-F

Table S6: Platinum SuperFI™ Green PCR Master Mix.

No.	Component	Volume
1	SuperFi™ Green PCR Master Mix	25 μL
2	5x GC enhancer	10 μL
3	10 μM Forward primer	1.5 μL
4	10 μM Reverse primer	1.5 μL
5	DHFR/T7S4 (pDT7S4 G13 M5) plasmid 10 ng/μL or mNeonGreen plasmid	1.5 μL
6	dd H2O	10.5 μL
Total of reaction		50 μL

Table S7: PCR (SuperFi™) program (same for all regardless of GOIs).

Initial denaturation	94°C	5 min	
Denaturation	94°C	45 s	3 cycles
Annealing	53°C	1 min	
Extension	72°C	5 min	
Denaturation	94°C	45 s	30 cycles
Annealing	65°C	1 min	
Extension	72°C	5 min	
Final extension	72°C	7 min	
Store	4°C	hold	

Table S8: GoTaq G2 Hot Start Green Master Mix DNA Polymerase.

No.	Component	Volume
1	GoTaq® G2 Hot Start Green Master Mix	5 μL
2	10 μM Forward primer	0.3 μL
3	10 μM Reverse primer	0.3 μL
4	DNA Template	2 μL
5	dd H2O	2.4 μL
Total of reaction		10 μL

Table S9: PCR conditions for GoTaq DNA Polymerase

Initial denaturation	98°C	2 min	
Denaturation	98°C	30 s	25-35 cycles
Annealing	Various*	30 s	
Extension	72°C	1 min/kb	
Final extension	72°C	5 min	/
Store	4°C/10°C	hold	/

***(5°C less than Tm temperature of each pair of primers)**

Table S10: List of primers designed and used in qRT-PCR

Primer name	Position	Sequence (5'UTR to 3'UTR)	Gene ID
LSD-2460	F	GGGTGGCGAAGACGAAAAAG	TgENT3
LSD-2461	R	CGCACCCCAACTTGACTCTT	
LSD-2464	F	GCAAATGAGCAAGGCCGATG	TgAT1
LSD-2465	R	GCACGCTAGCTGAAACATGG	
LSD-2472	F	GCGAAGGTTTCCCAGCTTAC	TgENT2
LSD-2473	R	CGCGACCGTTTCTTCTGTAG	
LSD-2093	F	TGTGGCACTACCCTGGTTTC	TgENT1
LSD-2094	R	GGACAACCCTGCTCAACACT	
LSD-715-Catalase	F	AGATCCTGACTACGCGGTTC	*Toxoplasma* internal control Catalase
LSD-716-Catalase	R	AGTTTTCCGACCGGGATGAG	
GPI8	F	GGCTGTCATTGTCTCCTCCT	*L. mexicana GPI8*
GPI8R	R	GTACATGGTAAGCGCATTGG	
